# Integrative network analysis identifies key genes and pathways in the progression of hepatitis C virus induced hepatocellular carcinoma

**DOI:** 10.1186/1755-8794-4-62

**Published:** 2011-08-08

**Authors:** Siyuan Zheng, William P Tansey, Scott W Hiebert, Zhongming Zhao

**Affiliations:** 1 Department of Biomedical Informatics, Vanderbilt University Medical Center, Nashville, TN 37232, USA; 2 Department of Cell and Developmental Biology, Vanderbilt University Medical Center, Nashville, TN 37232, USA; 3 Department of Cancer Biology, Vanderbilt University Medical Center, Nashville, TN 37232, USA; 4 Vanderbilt-Ingram Cancer Center, Vanderbilt University Medical Center, Nashville, TN 37232, USA; 5 Department of Biochemistry, Vanderbilt University Medical Center, Nashville, TN 37232, USA; 6 Cold Spring Harbor Laboratory, Cold Spring Harbor, NY 11724, USA

## Abstract

**Background:**

Incidence of hepatitis C virus (HCV) induced hepatocellular carcinoma (HCC) has been increasing in the United States and Europe during recent years. Although HCV-associated HCC shares many pathological characteristics with other types of HCC, its molecular mechanisms of progression remain elusive.

**Methods:**

To investigate the underlying pathology, we developed a systematic approach to identify deregulated biological networks in HCC by integrating gene expression profiles with high-throughput protein-protein interaction data. We examined five stages including normal (control) liver, cirrhotic liver, dysplasia, early HCC and advanced HCC.

**Results:**

Among the five consecutive pathological stages, we identified four networks including precancerous networks (Normal-Cirrhosis and Cirrhosis-Dysplasia) and cancerous networks (Dysplasia-Early HCC, Early-Advanced HCC). We found little overlap between precancerous and cancerous networks, opposite to a substantial overlap within precancerous or cancerous networks. We further found that the hub proteins interacted with HCV proteins, suggesting direct interventions of these networks by the virus. The functional annotation of each network demonstrates a high degree of consistency with current knowledge in HCC. By assembling these functions into a module map, we could depict the stepwise biological functions that are deregulated in HCV-induced hepatocarcinogenesis. Additionally, these networks enable us to identify important genes and pathways by developmental stage, such as *LCK *signalling pathways in cirrhosis, *MMP *genes and *TIMP *genes in dysplastic liver, and *CDC2*-mediated cell cycle signalling in early and advanced HCC. *CDC2 *(alternative symbol *CDK1*), a cell cycle regulatory gene, is particularly interesting due to its topological position in temporally deregulated networks.

**Conclusions:**

Our study uncovers a temporal spectrum of functional deregulation and prioritizes key genes and pathways in the progression of HCV induced HCC. These findings present a wealth of information for further investigation.

## Background

Hepatocellular carcinoma (HCC) is the third most common cause of cancer mortality in the world [1] and its incidence has been increasing in North America, Europe and Japan [2-5]. A recent study reported that approximately half of the observed increase in HCC is due to hepatitis C virus (HCV) infection, whereas the incidence of HCC related to other risk factors such as hepatitis B virus (HBV), alcoholic liver diseases or idiopathic cirrhosis has remained stable [6].

Like other etiological factors such as HBV, HCV induced HCC undergoes distinct histopathological stages, including chronic hepatitis, cirrhosis, dysplasia and eventually HCC [7]. Some genes were found to play critical roles in these processes, including *MMP9, TIMP1 *and *STAT1 *[8-11]. However, the spectrum of temporal pathway deregulation has rarely been studied using a systematic framework.

An approach for the examination of molecular events accompanying HCV related HCC progression is to leverage genome-wide technologies to search for deregulated genes and pathways in each pathological stage. Despite the increasing use of next generation sequencing in cancer studies [12,13], microarray gene expression is still widely applied as a mature and cost efficient technology [14,15]. For example, we recently identified progressively silenced genes in liver neoplasm transformation [16] and studied the functional roles of *HDAC3 *and its cofactor *NCOR1 *[17] in HCC using microarray data. In another recent study, 75 tissue samples representing stepwise HCV induced carcinogenesis from normal liver to HCC were analyzed using the Affymetrix Human Genome U133 plus 2.0 array platform, which identified gene signatures reflecting the pathological progression of the disease at each stage [3].

In this study, we applied a network-based approach to learn the specific molecular events underpinning the development of HCV induced HCC. Instead of comparing the gene expression profiles of two consecutive stages, we overlaid gene expression data with protein interaction networks (PIN) and identified representative subnetworks for each pathological stage. We focused on five stages including normal (control) liver, cirrhotic liver, dysplasia, early HCC and advanced HCC. Our resulting networks display the current biological knowledge regarding hepatocellular carcinogenesis and malignant transformation. We also found *CDC2 *(alternative symbol *CDK1*, encoding cyclin-dependent kinase 1) to be a critical gene in the continuous deregulation of the cell cycle in HCC progression.

## Methods

### Data collection

Gene expression data was downloaded from Gene Expression Omnibus (GEO) database [18]. Data set GSE6764 [3] was used to identify networks in this study. This data set includes 75 samples, including 8 distinct pathological stages, but no other clinical information is available for these samples. We excluded 3 samples from cirrhotic liver tissue of patients without HCC. To increase statistical power, we combined low-grade dysplastic nodules and high-grade dysplastic nodules as a dysplastic group, early HCC and very early HCC as an early HCC group, and advanced HCC and very advanced HCC as an advanced HCC group. As a result, 5 groups were included in our analysis, i.e., normal, cirrhosis, dysplasia, early HCC and advanced HCC. Since this data set had been already normalized when it was submitted to GEO, no more normalization was performed in our analysis. For genes with more than one probe set in the array platform, we used the maximal value in each sample to collapse those probe sets. Protein interaction data was downloaded from the Protein Interaction Network Analysis (PINA) platform [19]. As of 3/4/2010, the PINA platform contained 10,650 unique nodes and 52,839 edges. Each node represents a gene product (i.e., protein encoded by the gene) and each edge represents an interaction between the two linked nodes. To verify our results, we downloaded another independent microarray gene expression dataset, GSE14323 [15] from GEO. This dataset includes compatible normal and cirrhotic tissue samples, which we used to verify our normal-cirrhosis network.

The HCV-host protein interaction data was downloaded from the Hepatitis C Virus Protein Interaction Database [20] as of 7/10/2011. This database manually curated 524 non-redundant HCV protein and host protein interactions from literatures. A total of 456 human proteins were catalogued.

### Algorithm

To construct a network for each stage, we weighted each node in the protein interaction network by their expression fold changes (absolute value, log2 scale) between consecutive groups and obtained a node-weighted protein interaction network for each stage. We then ranked the genes by their weights and selected the top 500 genes as seed genes. That is, we obtained a list of 500 deregulated genes for each pair of consecutive stages (normal-cirrhosis, cirrhosis-dysplasia, dysplasia-early HCC, and early HCC-advanced HCC). We tested different numbers of top ranked genes as seeds, and the resulting networks were similar (data not shown). These genes were mapped to the network and used to extract a vertex-induced subnetwork, referred to as the seed network, from the stage specific network. It is worth noting that in practice these 500 genes may not be all present in the human interactome (i.e., PINA network). Therefore, only genes mapped in the whole human interactome were used as seeds. The following process of network query employs an iterative algorithm to expand the seed network, as was similarly done in our recent work on dense-module searching of genetic association signals (dmGWAS algorithm) from the genome-wide association studies (GWAS) [21]. The first step is to find the neighborhood node of maximum weight within a shortest path distance *d *to any node of the seed network. We chose *d *= 2 considering that the average node distance in the human protein interaction network is approximately 5 [22]. If the addition of the maximum weight neighborhood node yields a score larger than a certain criterion, the addition is retained and thus the network expands. This process iterates until no additional node meets the criterion, thus, iteration terminates. In each iteration, the seed network is scored by the average score of all nodes in the current network. Incorporation of a new node must yield a score larger than *S_net _*× (1 + *r*) where *r *is the rate of proportion increment. To obtain a proper *r *value, we set *r *from 0.1 to 2 with a step size 0.1 to assess the performance of subnetwork construction. For each *r *value, we ran the searching program and calculated the score of the resulting network. The *r *value leading to the first maximal network score was used as the final value of *r*. To avoid local optimization, median filtering was applied to smooth the score curve. According to our empirical observation, setting the maximum *r *to 2 is sufficient because scores are maximized before this value is reached (see additional file 1). The network was further refined by removing any component with less than 5 nodes so that we could prioritize more informative interacting modules. Eventually we identified 4 networks, named the Normal-Cirrhosis network, Cirrhosis-Dysplasia network, Dysplasia-Early HCC network and Early-Advanced HCC network. The first two networks are indicated as precancerous networks, whereas the latter two as HCC cancerous networks.

### Statistical significance test

We assessed network score significance with two tests. 1) We permuted the gene expression matrix by randomly swapping class labels. For genes in the 4 identified networks, we calculated gene weights from the random expression matrix and then determined a network score from these random gene weights. Statistical significance, denoted *P*_rand_, was computed as the proportion of random scores that are larger than or equal to the real score. Permutation trials were conducted over 1,000 iterations. 2) We permuted gene labels on the network so as to disrupt the correlation of gene weights and interactions. Then, we used the same seed genes to identify counterpart networks with identical procedures. We compared real network scores with the counterpart network scores to obtain *P*_perm_. The permutation trials were then conducted 100 times. We also tested the significance of topological structure in these networks. For each network, we generated 1,000 background networks with the Erdos-Renyi model [23,24]. Every background network has the same number of nodes and edges as the real network. We compared clustering coefficients of real networks with the background networks to obtain *P*_topo_.

### Enrichment analysis

We conducted functional enrichment analysis for the networks based on Gene Ontology (GO) Biological Process terms [25,26]. Enrichment significance was determined by analyzing a hypergeometric distribution as described previously [27]. *P *values were then corrected for false discovery rate (FDR). Gene sets containing less than 5 genes overlapping with the network were removed from the analysis. In our HCC module map, GO terms with an FDR-adjusted *P*-value of less than 0.05 in at least one network were retained.

## Results

### Overview of the networks and network connections

**Figure 1:**
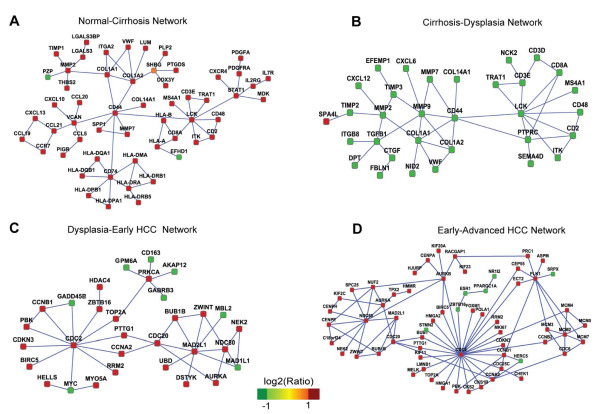
**Giant component of each stage specific network**. A, Normal-Cirrhosis network. B, Cirrhosis-Dysplasia network. C, Dysplasia-Early HCC network. D, Early-Advanced HCC network. Nodes in the network represent genes and edges represent their interactions. Node colour is scaled to gene expression fold change between two consecutive stages.

Following the sequence of normal, cirrhosis, dysplasia, early HCC and advanced HCC, we identified a representative network for each stage (Figure 1 for major component, Table 1). The full networks are provided in additional file 2. These networks are highly significant in terms of both score and topological structure measurements (no random case outperformed the real case, *P*_rand _< 1.0 × 10^-3^, *P*_perm _< 1.0 × 10^-2^, *P*_topo _< 1.0 × 10^-3^), which can be explained by a high proportion of differentially expressed genes (DEG) and hub proteins in the networks. Here, a hub protein is defined to have more than 5 protein interactions in those stage-specific networks. On average, DEGs account for 92.2 (± 5.4)% of nodes. Hub proteins occupy only 14.8 (± 3.6)% of the network nodes but are involved in 67.4 (± 10.0)% of associations. The existence of these hubs suggests network architecture being different from that of random networks and implicates potential modules of interest in these networks. Modules in biological networks often represent molecular complexes and pathways [24,28] which are the main objects of research in this study.

**Table 1:** Summary of the four networks

Network	#Genes	#Interactions	#DEGs*	#Hub interactions^†^	Hub gene^‡^
Normal to cirrhosis	55	67	53 (96.3%)	42 (62.7%)	*LCK, CD44, CD74, COL1A2, MMP2, VCAN, STAT1, COL1A1*

Cirrhosis to dysplasia	38	50	37 (97.4%)	35 (70.0%)	*LCK, CD44, MMP9, COL1A1, PTPRC, MMP2, TGFB1*

Dysplasia to early HCC	60	65	53 (88.3%)	37 (56.9%)	*CDC2, MAD2L1, PRKCA, CDC20, NDC80, UBQLN4*

Early to advanced HCC	68	98	59 (86.8%)	79 (80.6%)	*CDC2, NDC80, PLK1, MCM2, AURKB, CCNA2, CDC20, AURKA, CCNB1, NUF2, MMP9*

*Differentially expressed genes (DEGs) were defined as genes with fold change ≥ 2 or ≤ 0.5 and student t test *P *value ≤ 0.01.

^†^Hub interactions refer to the total number of interactions involving hub genes.

^‡^Hub genes were defined to have at least 5 interactions in each network.

**Figure 2:**
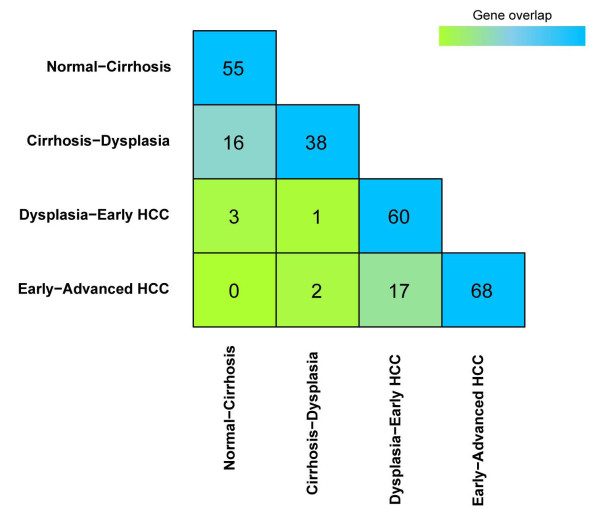
**Comparison of network comprising genes**. Numbers in the cell represent the number of overlapped genes in two networks. Colours are scaled according to the proportion of overlaps.

Although the four networks were identified independently, they have connections in terms of included proteins and interactions. As shown in Figure 2, the Normal-Cirrhosis network, which consists of 55 proteins, and Cirrhosis-Dysplasia network, which consists of 38 proteins, have 16 proteins in common, while the Dysplasia-Early HCC network (60 proteins) shares 17 proteins with Early-Advanced HCC network (68 proteins). It is important to note that precancerous networks (Normal-Cirrhosis, Cirrhosis-Dysplasia) and cancerous networks (Dysplasia-Early HCC, Early-Advanced HCC) only have marginal overlaps. This poor overlap suggests a dramatic difference of deregulation in cancerous and precancerous liver tissues.

### Verification of the representative network

**Figure 3:**
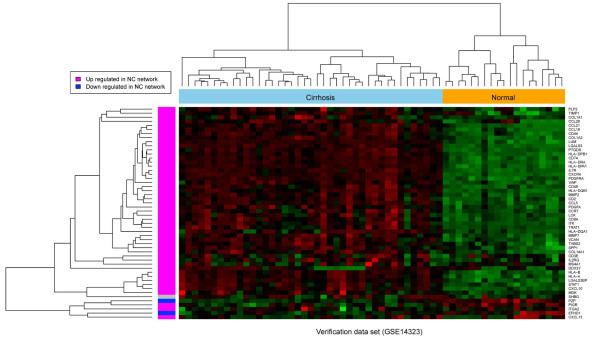
**Verification of Normal-Cirrhosis (NC) network in an independent data set**. Row colour bar indicates expression changes of NC genes in the experiment data. Pink red denotes up regulation in cirrhosis, blue denotes down regulation in cirrhosis. Column colour bar indicates phenotype. Orange denotes normal samples and sky blue denotes cirrhotic samples.

There are two possible ways for verification. One is to verify the robustness of expression patterns of the network genes and the other is to verify the robustness of the searching strategy. Due to the heterogeneity of available expression data on HCV induced HCC, we could not find a single independent dataset including clinical stage annotation to the extent of our experimental data. However, it is feasible for us to use matched data for the verification of specific networks. We used gene expression data from GSE14323 [15] to verify our Normal-Cirrhosis network. This data set includes normal, cirrhotic, and HCC tissue samples. To verify the expression patterns of the Normal-Cirrhosis network genes, we mapped the genes to this dataset. As shown in Figure 3, 94.2% of the Normal-Cirrhosis network genes display consistent expression changes in this additional data set. To verify the robustness of the searching approach, we used GSE14323 to identify Normal-Cirrhosis network following identical procedures. We found that 58.2% of the original Normal-Cirrhosis network genes are present in the verification network. Most hub proteins identified in original network are also hubs (degree > 5) in the verification network, including CD44, CD74, VCAN and MMP2. This high consistency indicates the reproducibility and reliability of the Normal-Cirrhosis network. Although the other 3 networks could not be verified due to the lack of compatible data, the case of the Normal-Cirrhosis network demonstrates that our approach can capture reproducible networks from gene expression data.

### Comparison of networks with HCV interacting proteins

All four networks comprise HCV binding proteins, as was summarized in Table 2. It is interesting to note that hub proteins are usually targeted, such as LCK, STAT1 and VCAN in Normal-Cirrhosis network, LCK in Cirrhosis-Dysplasia network, CDC2 and NDC80 in Dysplasia-Early HCC network and Early-Advanced network. HCV protein NS3 and NS5A seem to be actively involved in these interactions. The non-structure protein NS3 is a processive DNA helicase [29] and was suggested to associate with cancer related pathways such as Notch pathway [30], caspase 8 induced apoptosis [31], etc. NS5A was reported to play functional roles in immune invasion and carcinogenesis [32,33]. In a proteomic study, they were shown to co-regulate focal adhesion in human cells [34]. Our results implicate that these virus proteins could deregulate the core cellular functions, e.g., immune responses and cell cycle, by interacting directly with the hub proteins in the molecular network. We speculate that such a hub-targeting mechanism may represent a more effective approach for viruses to invade host's cellular machineries.

**Table 2:** Interactions between HCV proteins and network proteins

HCV protein	Normal-Cirrhosis	Cirrhosis-Dysplasia	Dysplasia-Early HCC	Early-Advanced HCC
CORE	HLA-A, STAT1, VWF, DDX3Y	VWF	-	-

NS5A	LCK	LCK	CDC2	CDC2

NS3	VCAN	FBLN1, EFEMP1, NID2, CTGF	NDC80	NDC80

NS4A	-	-	MT2A	-

p7	-	-	UBQLN4	LMNB1

The HCV-host protein interaction data was retrieved from the Hepatitis C Virus Protein Interaction Database [20] as of 07/10/2011.

### Network functions suggest molecular events in HCC progression

**Figure 4:**
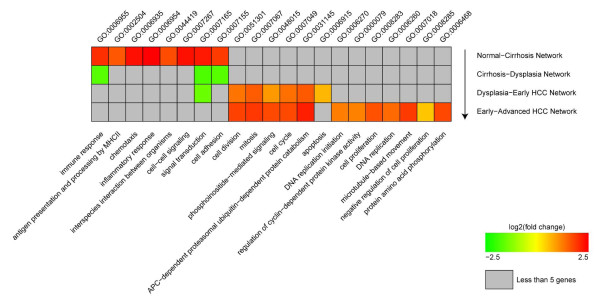
**Functional enrichment of the networks to Gene Ontology terms of Biological Process**. Colours were scaled by the average fold changes of that gene set.

To understand these networks holistically, we applied functional enrichment analysis based on the Gene Ontology resource [25]. In total, 21 significant biological processes (FDR ≤ 0.05) were prioritized. Distribution of these processes varies for each particular network. We compiled these processes into a single array, referred to as the HCC module map, to learn the deregulation spectrum of HCC progression (Figure 4).

In the Normal-Cirrhosis network, which corresponds to early stages of HCV infection, immune response, MHC Type II activity, inflammatory response and cell-cell signalling are enriched, indicating that a systematic protection mechanism is activated in response to HCV. Some cell adhesion genes are also up regulated in cirrhotic livers. These protection mechanisms seem impaired in dysplastic livers, as is suggested by the down regulation of immune response in our Cirrhosis-Dysplasia network. Moreover, cell adhesion and signal transduction are also down regulated, indicating the presence of more invasive and migratory hepatocytes in dysplastic nodules [35].

A clear pattern in HCC networks (Dysplasia-Early HCC network, Early-Advanced HCC network) is that many more pathways are deregulated in the advanced HCC network while the majority of pathways prioritized in the early HCC network remain continuously up regulated. These consistently deregulated pathways may play critical roles in the transition from early HCCs to more advanced stages considering their correlation to the temporal order of HCC progression. Represented in this group were cell cycle, cell division, and mitosis related pathways (Figure 4).

**Figure 5:**
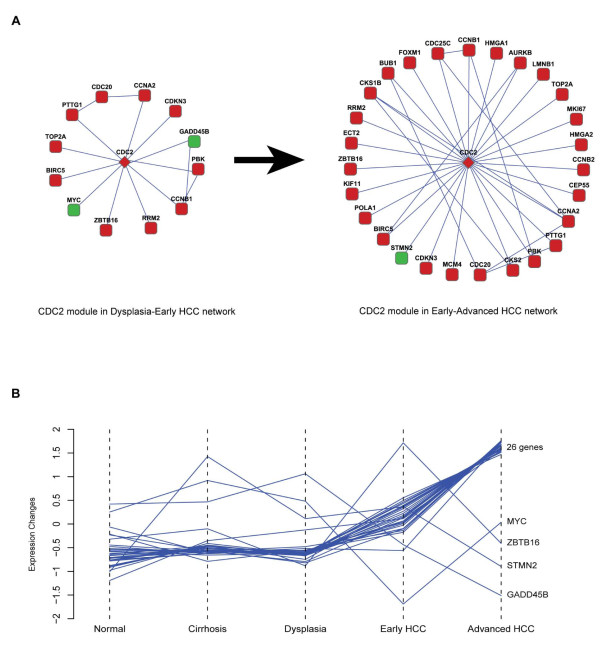
**Evolution of CDC2 module in HCC progression**. A, module overview in Dysplasia-Early HCC network and Early-Advanced HCC network. B, expression profiles of CDC2 module genes in five pathological stages.

Despite the consistency, the HCC module map discloses a discrepancy in the Dysplasia-Early HCC network and Early-Advanced HCC network related to apoptosis. We examined apoptosis genes in these networks and found that gene *ZBTB16 *(encoding zinc finger and *BTB *domain containing 16, also known as *PLZF*) has opposing expression patterns. That is, *ZBTB16 *is up regulated in early HCC, and then down regulated in advanced HCC. One function of *ZBTB16 *is to prevent cell cycle progression [36] and suppress solid tumorigenesis [37]. The expression pattern of *ZBTB16 *revealed in our study is consistent with those results and indicates a role for loss of *ZBTB16 *expression in HCC progression. Furthermore, strong negative correlation of its expression pattern with *c-Myc *was observed (Figure 5B), indicating a possible regulation mechanism between these two genes. Regulation of *c-Myc *by *ZBTB16 *was previously reported in acute promyelocytic leukemia cell line [36]. In HCC, for the first time to our best knowledge, we show this regulation sustains in cancerous stage, suggesting that it might be a universal mechanism in carcinogenesis.

### Networks prioritize genes and pathways in HCC progression

One advantage of the network approach is that networks contain interaction information and visualization of gene interactions provides an intuitive way to explore gene functions in context. We present giant components (the biggest connected subgraph) of the 4 networks in Figure 1. These giant components account for 100.0%, 84.2%, 51.7% and 91.2% of the network nodes, respectively.

In the Normal-Cirrhosis network there are some apparent module structures such as the MHC II complex (major histocompatibility complex, class II), LCK (lymphocyte-specific protein tyrosine kinase) signalling module, chemokine gene module, etc. MHC II molecules are antigen presenting proteins involved in cell-mediated immunity, while LCK is a key regulator of T cell activation and development [38-40]. Their up-regulation suggests enhanced adaptive immunity responses, which partially determine the outcome of HCV infection [41]. Signalling molecule STAT1 was also a hub protein in this network.

Adaptive immunity appears depressed considering the overall down regulation of the LCK signalling module in our Cirrhosis-Dysplasia network. Another module in this network comprises extracellular matrix (ECM) regulatory and constituent genes, such as *MMP*s, *TIMP2, COL1A1*, and *COL1A2. TGF-beta 1*, which was reported to increase ECM production [42], is also included in this module. Deregulation of this ECM related module is strongly indicative of the aberrant morphology of dysplastic nodules, which are regarded as primary precancerous lesions [14].

Unlike the LCK signalling module that shows opposing expression patterns in precancerous networks, the CDC2 centered cell cycle module and MAD2L1 and NDC80 centered spindle checkpoint signalling module preserve their expression patterns in HCC networks. Also the Early-Advanced HCC network includes a minichromosome maintenance complex module and PLK1 (Polo like kinase 1) centered module, which are involved in cell division [43,44]. Because of its role in cell proliferation, *PLK1 *has been proposed as a potential therapeutic target in many cancers [45,46].

An important consensus of the HCC networks is hub protein CDC2. CDC2, also known as CDK1 (cyclin dependent kinase 1), is a key regulatory kinase of the cell cycle [44]. We compared CDC2 modules from the Dysplasia-Early HCC network and Early-Advanced HCC network, and found that more deregulated genes are involved in the latter (Figure 5A). Expression pattern analysis indicates that the majority (86.7%) of the genes whose encoded proteins interact with CDC2 in HCC networks have continuously increasing expression during carcinogenesis (Figure 5B). This pattern indicates that this module is evolving towards a more deregulated form in both size and extent during the progression of HCC. A previous study has shown that the HCV core protein perturbs both G1/S and G2/M phases of cell cycle by up regulating the expression and activity of cyclin B1-CDC2 complex [47]. Our network analysis confirmed that finding. Furthermore, we show that many more CDC2 interacting genes have concordant expression profiles in HCC. This concordance suggests that there might be some common regulatory mechanisms controlling the behavior of those closely associated genes.

Although it remains unclear whether this module is one of the driving forces for HCC malignancy, our results implicate that by drug interference to this module, HCC progression could be prevented to some extent. Considering that CDC2 is a hub protein in the network, its inhibition might be an effective way for functional interference to this module. Further, CDC2 is a kinase, which is a major druggable protein class [48,49]. A pilot study reported that inhibition of CDC2 could decrease tumor growth and is a potential therapy for hepatoblastoma tumor and some other tumors [50]. Our study, which provides further support for this treatment strategy, suggests that a similar strategy may be applied to HCV induced HCC for clinical therapy.

## Discussion

In this study, we developed an integrative network approach and applied it to study deregulated events in HCV induced HCC. Unlike common pathway resources such as KEGG biochemical pathways [51] or Gene Ontology [25], we integrated microarray data with high throughput protein-protein interaction data and searched for deregulated networks during each pathological stage. Compared to pathways, networks give more explicit protein interactions and provide flexible setting of gene sets for investigation and, thus, facilitate generation of novel hypotheses.

Employing this approach, we attempted to dissect the progression of HCV induced HCC. Findings in this work not only confirmed many previous reports, but also provided many novel and important insights. For two examples, immune response was over-activated in cirrhotic livers but then impaired in dysplastic nodules; continuous up regulation of cell cycle and related processes such as mitosis in HCC were detected by our approach. Pivotal genes involved in these processes were highlighted, including *LCK, MMP *genes, *CDC2*, etc. Many cancer-related genes were also observed such as *CCNA2, AURKA, BIRC5 *and *GADD45B*. We further prioritized the CDC2 network module due to its evolutionary pattern in HCC progression. To our best knowledge, this is the first time that this module is highlighted in a systematic manner in HCC studies.

Our data suggests that deregulations of these cellular processes may result from direct HCV protein interactions with the hub proteins in the molecular networks. The most noticeable virus proteins in those interactions are HCV non-structure protein NS3 and NS5A, both of which were suggested to be potentially important for liver tumorigenesis [30-33]. Our finding that the HCV proteins target the identified networks provides a supportive argument on the utility and effectiveness of integrative network approach to studying the molecular mechanisms underlying complex diseases or traits.

There are several limitations in this study. First, the complete human interactome data is still unavailable by now, even though both the quantity (number of protein-protein interaction pairs) and the quality (curation, experimentally verified) of the data have been greatly improved during the past years [19]. Second, findings and conclusions in this work are derived from computational analysis and then are largely verified by literature survey. Further functional and biological validation is needed. Moreover, expression deregulation revealed by microarrays may be limited because many genomic alterations occur on different levels such as post-transcriptional and post-translational levels and metabolic level. It would be interesting to integrate those data in future studies in order to reveal a comprehensive landscape of HCC pathogenesis at the molecular level.

Despite those limitations, our approach renders a model to extract information from high throughput genomic experiments. Our results show that such an integrative method is promising to decipher complex diseases, especially in front of current genome biotechnologies such as microarray and whole transcriptome sequencing (RNA-Seq by next generation sequencing).

## Conclusions

We developed an integrative network approach and applied it to study deregulated events in HCV induced HCC. Instead of comparing the gene expression profiles of two consecutive stages, we overlaid gene expression data with protein interaction networks to identify representative subnetworks for each pathological stage and deregulated subnetworks in disease progression. Our study uncovered a temporal spectrum of functional deregulation and prioritized key genes and pathways in the progression of HCV induced HCC. Among them, *CDC2 *was found to be a critical gene in the continuous deregulation of the cell cycle in HCC progression. These findings present a wealth of information for further investigation.

## Competing interests

The authors declare that they have no competing interests.

## Authors' contributions

SZ, ZZ, SWH and WPT conceived and designed the experiments. SZ carried out the data analysis. SZ, ZZ, SWH and WPT drafted the manuscript. All authors read and approved the final manuscript.

## Pre-publication history

The pre-publication history for this paper can be accessed here:

http://www.biomedcentral.com/1755-8794/4/62/prepub

## Supplementary Material

Additional file 1 **Relationship of stepwise γ values and network scores**. Red node is selected as cut-off for network identification. A, Normal-Cirrhosis Network; B, Cirrhosis-Dysplasia Network; C, Dysplasia-Early HCC Network; D, Early-Advanced HCC Network. Click here for file

Additional file 2 **Stage specific networks**. Nodes represent gene products and edges represent their interactions. Colour is scaled according to gene expression fold change between two consecutive stages. Click here for file
