# Supplementary figures and images for: Integrative network analysis identifies key genes and pathways in the progression of hepatitis C virus induced hepatocellular carcinoma
Source: BMC Med Genomics. 2011 Aug 8;4:62. doi: 10.1186/1755-8794-4-62 (PMC3212927; doi:10.1186/1755-8794-4-62)

Figure 1S

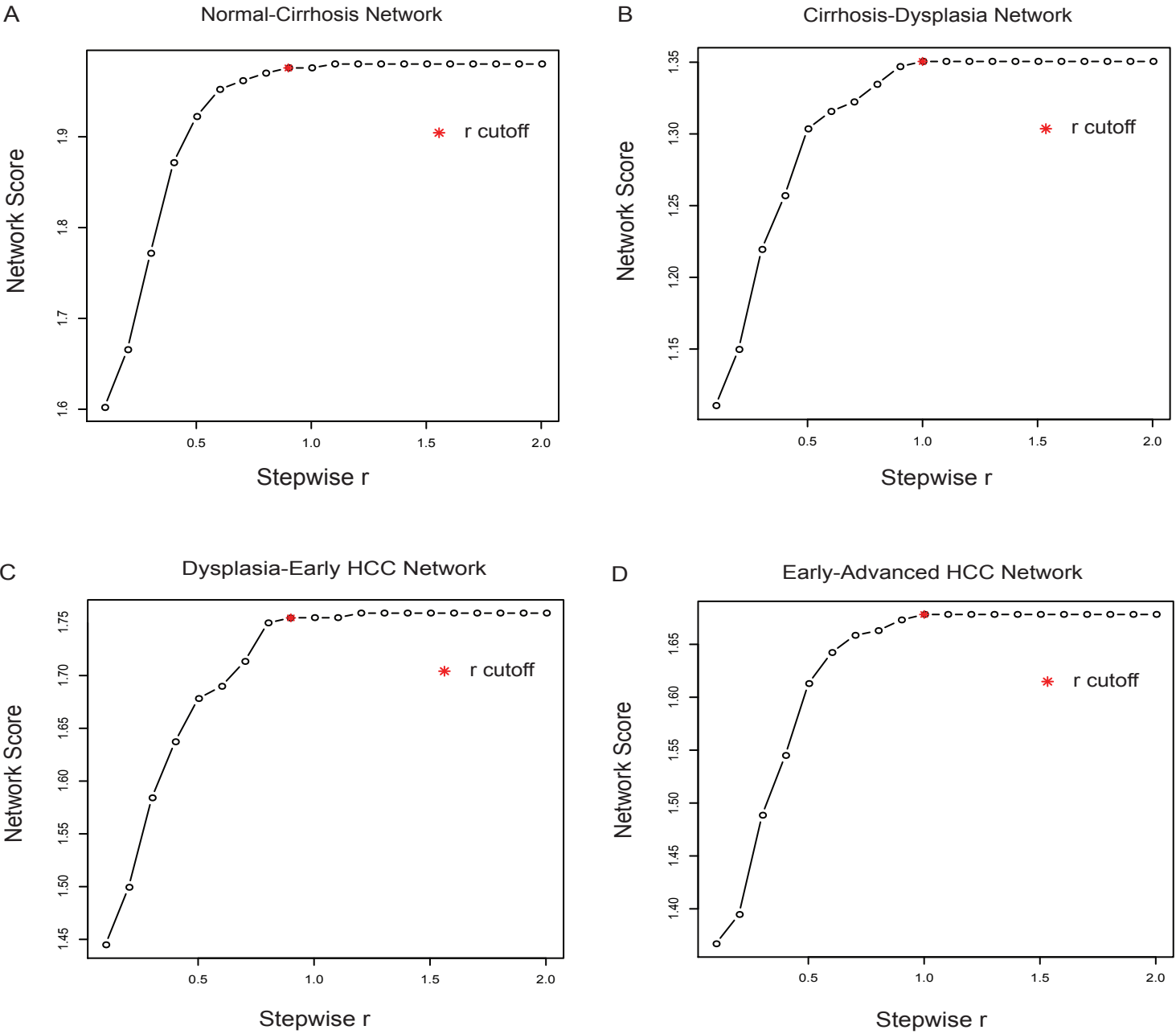

Supplement: Additional file 1 — Relationship of stepwise γ values and network scores. Red node is selected as cut-off for network identification. A, Normal-Cirrhosis Network; B, Cirrhosis-Dysplasia Network; C, Dysplasia-Early HCC Network; D, Early-Advanced HCC Network. [file 1755-8794-4-62-S1.PDF]
