# Supplementary material for: Integrative network analysis identifies key genes and pathways in the progression of hepatitis C virus induced hepatocellular carcinoma
Source: BMC Med Genomics. 2011 Aug 8;4:62. doi: 10.1186/1755-8794-4-62 (PMC3212927; doi:10.1186/1755-8794-4-62)

Figure 2S

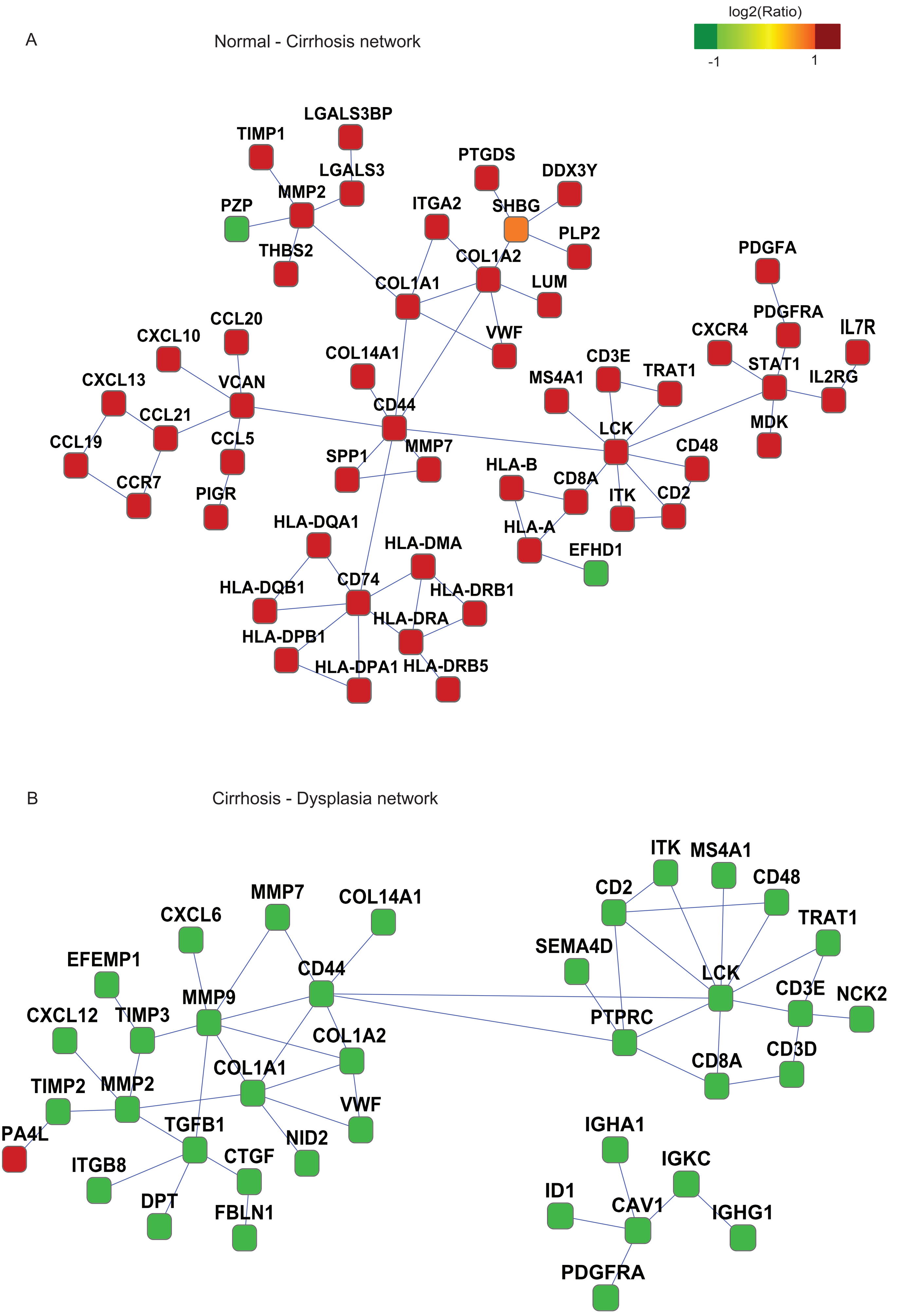
log2(Ratio)
-1
1
A
Normal - Cirrhosis network
LGALS3BP
TIMP1
PTGDS
LGALS3
DDX3Y
MMP2
PZP
ITGA2
SHBG
PLP2
THBS2
COL1A2
PDGFA
COL1A1
LUM
CCL20
PDGFRA
CXCL10
VWF
CXCR4
IL7R
COL14A1
CD3E
STAT1
CXCL13
VCAN
MS4A1
TRAT1
IL2RG
CCL21
CD44
CCL5
MDK
LCK
CCL19
SPP1
MMP7
CD48
HLA-B
CCR7
CD8A
PIGR
ITK
CD2
HLA-DQA1
HLA-A
HLA-DMA
EFHD1
CD74
HLA-DQB1
HLA-DRB1
HLA-DRA
HLA-DPB1
HLA-DPA1
HLA-DRB5
B
Cirrhosis - Dysplasia network
ITK
MS4A1
CD2
CD48
MMP7
COL14A1
CXCL6
TRAT1
SEMA4D
EFEMP1
CD44
LCK
CD3E
NCK2
MMP9
CXCL12
TIMP3
PTPRC
COL1A2
CD3D
COL1A1
CD8A
TIMP2
MMP2
VWF
IGHA1
TGFB1
HSPA4L
NID2
CTGF
IGKC
ITGB8
ID1
DPT
CAV1
IGHG1
FBLN1
PDGFRA

C

Dysplasia - Early HCC network

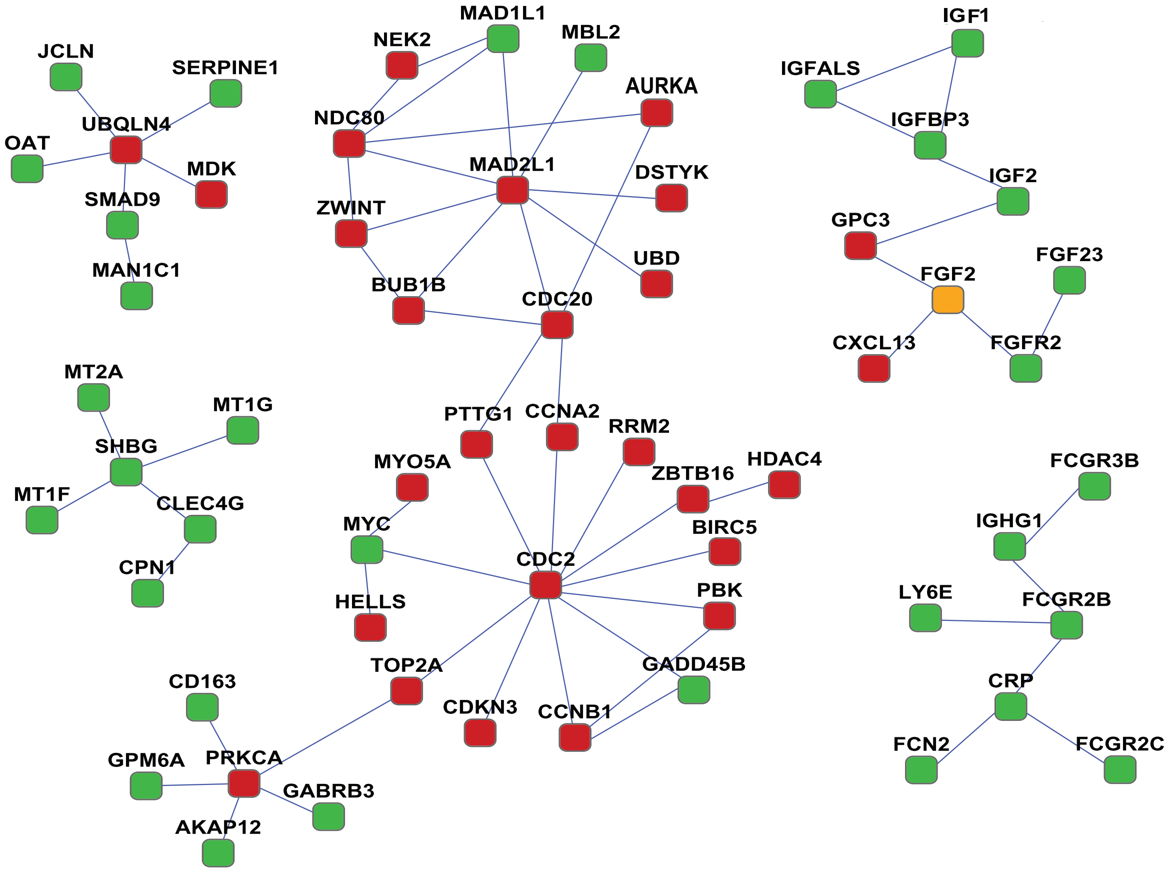

D

Early - Advanced HCC network

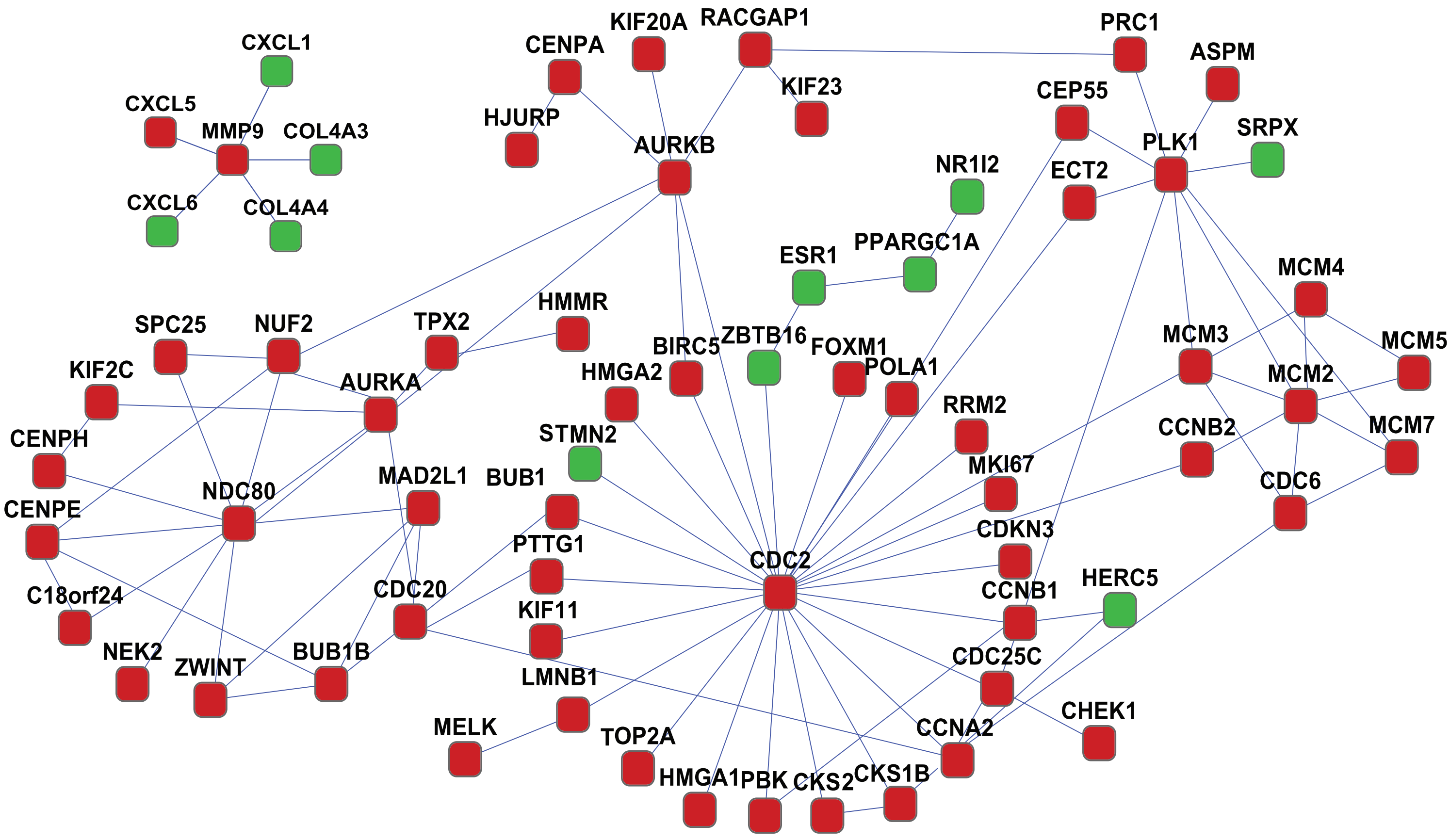

Supplement: Additional file 2 — Stage specific networks. Nodes represent gene products and edges represent their interactions. Colour is scaled according to gene expression fold change between two consecutive stages. [file 1755-8794-4-62-S2.PDF]
